# Antibiotic Prophylaxis in Instrumented Lumbar Spine Surgery: Cefazolin Outperforms Clindamycin Regardless of Duration

**DOI:** 10.3390/antibiotics14080830

**Published:** 2025-08-15

**Authors:** Zoltán Nagy, Dóra Szabó, Gergely Agócs, Konrád Szilágyi, Zsanett Rojcsik, József Budai, Zoltán Papp, Csaba Padányi, Loránd Erőss, László Sipos, Péter Banczerowski

**Affiliations:** 1 Clinic of Neurosurgery and Neurointervention, Semmelweis University, 1085 Budapest, Hungary; nagy.zoltan2@semmelweis.hu (Z.N.); sipos.laszlo.kornel@semmelweis.hu (L.S.); banczerowski.peter@semmelweis.hu (P.B.); 2 Department of Neurosurgery, Semmelweis University, 1085 Budapest, Hungary; 3 Institute of Medical Microbiology, Semmelweis University, 1089 Budapest, Hungary; 4 HUN-REN-SU Human Microbiota Research Group, 1052 Budapest, Hungary; 5 Department of Biophysics and Radiation Biology, Semmelweis University, 1094 Budapest, Hungary

**Keywords:** instrumented spine surgery, surgical site infection (SSI), superficial SSI, deep SSI, organ SSI, antibiotic prophylaxis, single dose antibiotic prophylaxis, prolonged antibiotic prophylaxis, 72 h antibiotic prophylaxis, cefazolin, clindamycin

## Abstract

**Background:** Surgical site infections (SSIs) are a significant postoperative complication in instrumented lumbar spine surgery, and the selection and duration of appropriate prophylactic antibiotics are key to their prevention. The aim of our study was to evaluate the effectiveness of various prophylactic antibiotics, primarily cefazolin and clindamycin, as well as the role of the duration of antibiotic prophylaxis in the development of SSI in instrumented lumbar spine surgeries through retrospective analysis. **Methods:** We performed a retrospective analysis of data from 915 patients who underwent instrumented lumbar spine surgery between 2016 and 2024 in a university center database. We examined the incidence of SSI according to the type of antibiotic used (cefazolin 1 g or 2 g, or clindamycin 0.6 g) and the duration of prophylaxis (single dose versus 72 h administration). We used the Fisher test and Welch test as a statistical analysis to examine the differences between SSI rates. **Results:** The incidence of SSI was 11.7%. We measured a significantly lower infection rate with cefazolin compared to clindamycin (OR = 0.45; 95% CI: 0.23–0.94; *p* = 0.0206), regardless of the duration of antibiotic administration. The 72 h cefazolin prophylaxis showed a slight but statistically insignificant advantage over single dose prophylaxis. The risk of SSI was significantly higher in multi-segment surgeries (*p* = 0.0005). **Conclusions:** Cefazolin is a more effective prophylactic antibiotic than clindamycin during instrumented lumbar spine surgery. The duration of antibiotic administration has less influence on the risk of SSI development; therefore, short-term, adequate-dose cefazolin prophylaxis is recommended, which also minimizes the risk of antimicrobial resistance and side effects.

## 1. Introduction

Surgical site infection (SSI) following spinal surgery is a serious postoperative complication. Several factors, such as surgical characteristics, for example, the posterior surgical approach, arthrodesis, and use of spinal implants, along with patient characteristics, e.g., patient age, BMI, diabetes mellitus, hypertension, duration of surgery, smoking habits, and blood loss during surgery, can significantly increase the risk of infection [1,2,3].

Over the past few decades, there has been a marked increase in the number of spinal fusion surgeries performed worldwide, accompanied by a decline in non-instrumented procedures [4,5]. Infection rates also vary according to the type of spine surgery: lumbar discectomy typically has an infection rate below 1%, while non-instrumented spinal fusions range from less than 1% up to 5% [6]. Instrumented spinal fusion is associated with a significantly higher risk of SSIs compared to non-instrumented surgeries, with reported infection rates ranging from 1.4% to 20%, depending on the surgical technique employed [7,8,9]. SSIs can range from superficial to deep incisional and organ/space infections. Deep or organ SSIs are common complications of instrumented spinal surgery associated with patient morbidity, poorer long-term outcomes, and higher healthcare costs [10]. The prevention of elevated infection risk for SSIs is a critical priority for spine surgeons, with systemic antimicrobial prophylaxis serving as an effective preventive measure.

Antimicrobial prophylaxis has become a standard practice in surgery to reduce the risk of postoperative infections. First-generation cephalosporins (e.g., cefazolin) are commonly used for preoperative prophylaxis in spine surgery due to their efficacy against common skin flora and favorable safety profile [11,12]. Alternatives such as clindamycin or levofloxacin are used in patients with allergies to beta-lactams [13]. The current guidelines recommend that systemic antibiotics should be administered within 30–60 min before the skin incision to ensure adequate tissue levels during surgery. The standard recommendation is to discontinue antibiotics within 24 h after spine surgery in uncomplicated cases. This approach helps to minimize risks of antimicrobial resistance, toxicity, unnecessary costs, and paradoxically, higher postoperative infection rates associated with prolonged antibiotic use [11,14].

Despite adequate antibiotic prophylaxis, the rate of SSIs following spinal surgery remains between 0.7% and 10%. In healthy patients, this rate ranges from 0.7% to 4.3%, while in patients with comorbidities, it ranges from 2.0% to 10%. Even the best antibiotic regimens currently in use have not been able to completely prevent SSIs [15].

Some recent studies suggest that extended, prolonged antibiotic prophylaxis may reduce the incidence of SSIs, particularly superficial infections, in instrumented spinal fusion surgeries [16,17]. However, evidence is mixed, and extended prophylaxis must be balanced against risks of adverse effects and antibiotic resistance. There is still ongoing debate regarding the optimal choice of antibiotic and the appropriate duration of its administration for different surgical procedures, especially in instrumented spine surgery.

Our aim was to evaluate retrospectively the impact of different preoperative antibiotic prophylaxis regimens on the development of SSIs in patients undergoing instrumented lumbar spine surgery between 2016 and 2024. Specifically, we aimed to determine the extent to which different types of antibiotics influence the development, incidence, and severity of SSIs during the study period. The additional purpose of the study was during the period between 2023 and 2024 to compare the single dose and 72 h cefazolin protocols’ effect on the development and severity of SSIs.

## 2. Results

### 2.1. Patients Data and Characteristics

Data from a total of 915 instrumented spine surgery cases were collected and analyzed during our study. The data were collected between 2016 and 2024, and the characteristics are shown in Table 1.

There was no significant difference in age, gender, or ratio of patients with diabetes mellitus between the non-infected and SSI groups. However, the BMI was slightly higher with a significant difference (*p* = 0.0081) in the SSI group.

In instrumented lumbar spine surgery, SSIs were documented in 107 of 914 cases (11.7%); however, 807 cases (88.3%) were free of infection in the study period between 2016 and 2024. Among the SSIs, the most common SSI was organ with 4.5% (41 infections out of 914 cases), followed by deep SSIs in 3.9% (36 infections out of 914 cases) and finally superficial SSIs with a 3.3% rate (30 infections out of 914 cases). Regarding surgical characteristics, there were significantly (*p* = 0.0005) more multi-segment surgeries involving three to seven segments in the SSI group (33.6%) compared to the non-infected group with a 13.2% rate.

However, using a minimally invasive technique decreased the occurrence of SSIs from 19.8% to 15%, but this reduction was not significant (*p* = 0.2959).

### 2.2. Antibiotic Prophylaxis Regimens and the Prevalence of SSIs

During the entire study period from 2016 to 2024, various regimens of preoperative antibiotic prophylaxis were employed (Table 2). Cefazolin was used as the standard prophylactic agent in a single dose of either 1 g or 2 g based on the BMIs of the patients. While the duration of antibiotic prophylaxis was not standardized during the entire study period, a 72 h regimen was limited to a small group of patients (n = 26) in 2023 and 2024, based on the operating surgeon’s preference. Considering that other pre- and postoperative conditions were consistent, the relationship between the incidence of SSIs and the type of antibiotic prophylaxis was analyzed.

**Figure 1:**
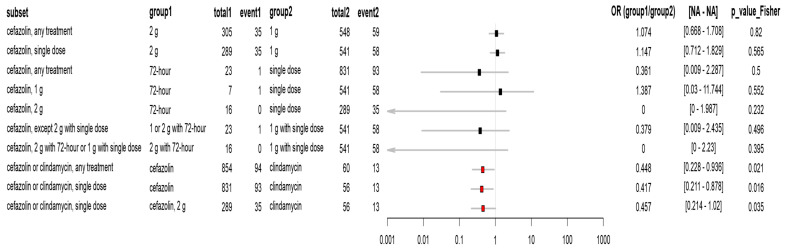
A forest plot showing the comparison of the different antibiotic prophylaxis regimens and the rate of SSIs for the 2016–2024 period.

Comparing the efficacy of each antibiotic protocol, a significant difference was found between the use of cefazolin and clindamycin. Compared with clindamycin, the use of cefazolin was associated with a significantly lower rate of SSIs (0.110 (cefazolin) vs. 0.217 (clindamycin); *p* = 0.0206), regardless of the duration of prophylaxis. This difference was even more pronounced when only single dose prophylaxis was compared (0.112 (cefazolin) vs. 0.232 (clindamycin); *p* = 0.0165) and was also significant when only cefazolin 2 g was compared with clindamycin (0.121 (cefazolin, 2 g) vs. 0.232 (clindamycin); *p* = 0.0352) (Figure 1).

In contrast, within-cefazolin comparisons, whether of dose (1 g vs. 2 g) or duration of prophylaxis (72 h perioperative versus single dose), did not show any statistically significant differences in incidence of SSI cases. Comparing 2 g and 1 g cefazolin did not yield significant differences in all treated groups (0.115 (2 g) vs. 0.108 (1 g); *p* = 0.8196), nor in the single dose groups (0.121 (2 g) vs. 0.107 (1 g); *p* = 0.5645). Similarly, the duration of prophylaxis (72 h extended versus different single doses) did not significantly affect the incidence of SSIs in either the overall population (0.043 (72 h) vs. 0.112 (single dose); *p* = 0.5001), 1 g (0.143 (72 h) vs. 0.107 (single dose); *p* = 0.5516), or 2 g (0.000 (72 h) vs. 0.121 (single dose); *p* = 0.2318) subgroups. Furthermore, the various combinations compared to a single dose of 1 g cefazolin—either a 2 g dose or 72 h extended use—did not show any significant difference (0.043 (1 or 2 g with 72 h) vs. 0.107 (1 g with single dose); *p* = 0.4963 in both cases) (Figure 1).

The forest plot illustrates the association between different antibiotic prophylaxis regimens and the incidence of surgical site infections (SSIs). Each row represents a comparison between the two treatment groups within a subset of the study population. The effect size is expressed as the odds ratio (OR), calculated as the odds of infection in group 1 divided by the odds in group 2. *p*-values were derived using Fisher’s exact test. Red squares indicate a significant effect.

### 2.3. Effect of Single Dose Antibiotic Prophylaxis—1 g Cefazolin, 2 g Cefazolin, and 0.6 g Clindamycin—On Rate of SSIs

**Figure 2:**
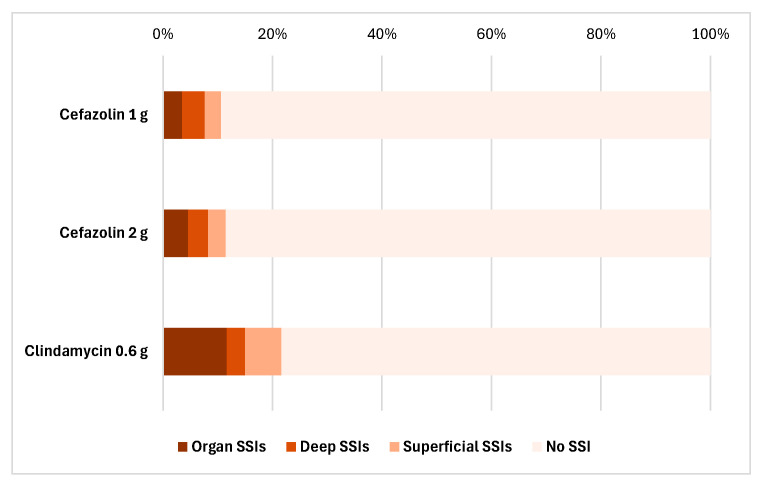
The distribution of superficial, deep, and organ SSIs after using cefazolin 1 g, cefazolin 2 g, and clindamycin 0.6 g as a single dose antibiotic prophylaxis.

In the case of single dose antibiotic prophylaxis, the highest rate of SSIs was observed with single dose clindamycin prophylaxis, at 21.67%. In contrast to that, SSI rates were lower with cefazolin usage: 10.6% with a 1 g dose cefazolin and 11.52% with a 2 g dose. In terms of the distribution of SSI severity, clindamycin prophylaxis was responsible for approximately twice as many deep (15%) and superficial (6.67%) infections as cefazolin 1 g (7.67% and 2.63%) or 2 g (8.23% and 3.29%) (Figure 2).

These results support the conclusion that cefazolin, especially when used at the appropriate dose, is a more effective and safer choice for surgical prophylaxis, while the use of clindamycin is associated with a significantly higher risk of SSIs.

**Figure 3:**
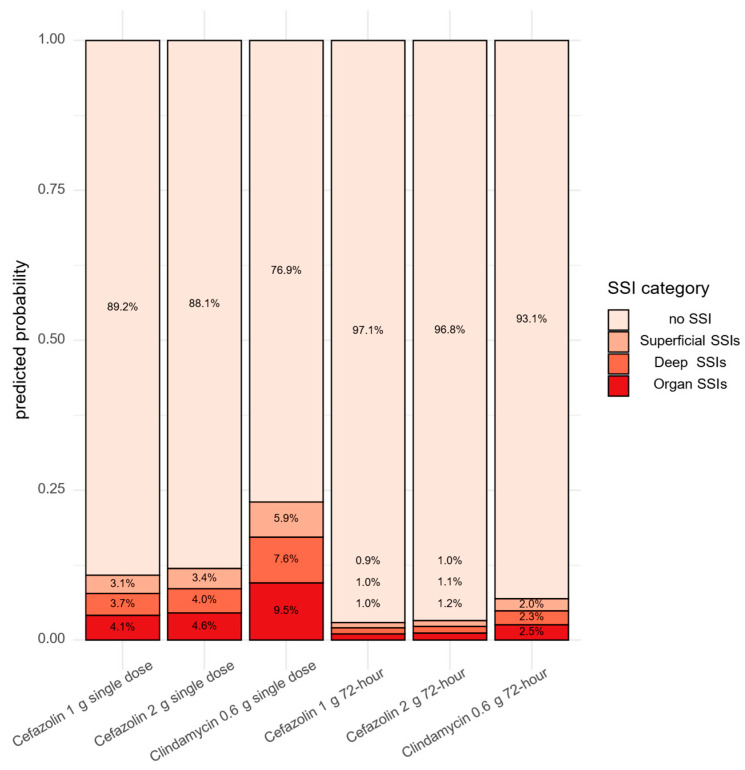
Predicted proportions of different SSI severities based on antibiotic type and dose regimen.

An ordinal logistic regression model with a cumulative link was employed to estimate the odds of more severe SSI as a function of the antibiotic–dosage category (cefazolin 1 g, cefazolin 2 g, clindamycin 0.6 g, 72 h) and the regimen category (single dose, 72 h) without their interaction. This model did not detect a significant difference between the regimens either: OR for 72 h vs. single dose: 0.25 (*p*-value: 0.1744). However, cefazolin turned out to be superior to clindamycin when the antibiotic–dosage categories were compared: OR of cefazolin 1 g versus cefazolin 2: 0.90 (*p*-value: 0.8800); OR of cefazolin 1 g versus clindamycin 0.6 g: 0.41 (*p*-value: 0.0231); and OR of cefazolin 2 g versus clindamycin 0.6 g: 0.45 (*p*-value: 0.0719). Based on this model, the proportions of different SSI severities could be predicted as shown in Figure 3.

### 2.4. Effect of Single Dose Versus 72 h Antibiotic Prophylaxis on SSI Occurance Between 2023 and 2024

To explore the potential differences in clinical outcomes between single dose and 72 h antibiotic prophylaxis, we conducted a focused analysis of cases with instrumented lumbar spinal surgery in the 2023 and 2024 period. During this period, twenty-six patients received 72 h antibiotic prophylaxis, among which twenty-two patients received cefazolin prophylaxis: seven patients received 1 g and fifteen patients received 2 g cefazolin. Among patients who received 72 h cefazolin prophylaxis, one case of deep surgical site infection (SSI) was documented. Furthermore, four patients received 72 h clindamycin prophylaxis; no SSIs were observed among them (Table 3).

During the same 2023 and 2024 period, as single dose prophylaxis, 100 patients received 1 g of cefazolin, 115 patients received 2 g of cefazolin, and 12 patients received single dose clindamycin. Altogether during this period, twenty-three SSIs cases (9%) were reported: in two patients, superficial SSIs occurred; in eleven patients, deep SSIs were observed; and in ten patients, organ SSIs developed. SSI rates varied across prophylactic regimens: 10.0% (10/100) in patients receiving single dose cefazolin 1 g, 7.8% (9/115) in those receiving single dose cefazolin 2 g, and 25.0% (3/12) in the clindamycin single dose group (Table 3).

**Figure 4:**
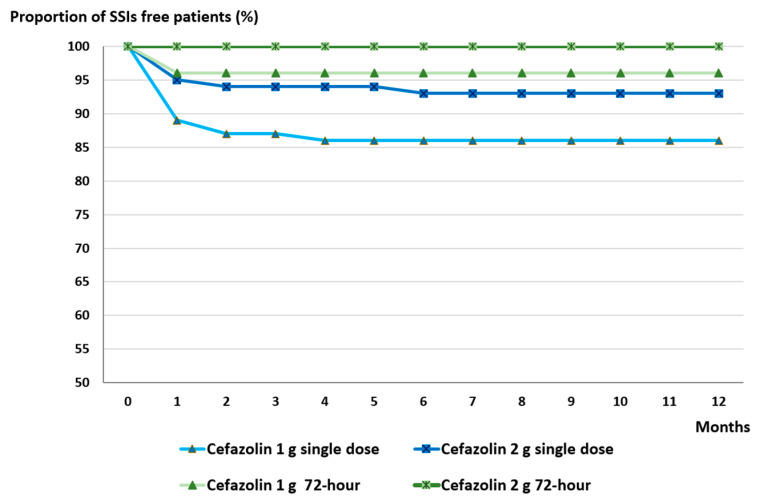
Proportion of SSI-free patients during one-year follow-up after instrumented spinal surgery using different cefazolin prophylaxis regimens.

We assessed the duration of the infection-free period following 1 g and 2 g cefazolin administration, both as single dose and 72 h prophylaxis, during a one-year follow-up. As shown in Figure 4, the 72 h regimen yielded more favorable outcomes: no SSIs were observed in the 2 g group and only one case occurred in the 1 g group. Single dose cefazolin (2 g and 1 g) followed in terms of effectiveness.

**Figure 5:**
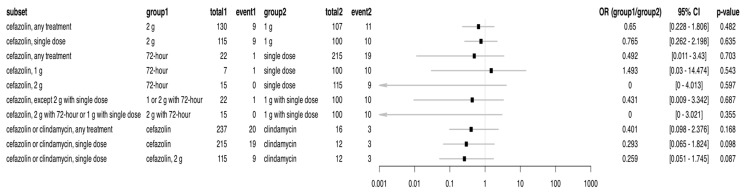
A forest plot showing the comparison of the different antibiotic prophylaxis regimens and the rate of SSIs for the 2023–2024 period.

The analyses in Figure 1 were conducted on the full dataset covering 2016–2024. It is important to note that before 2023, there was no 72 h prophylaxis regimen in place. Analyses on the restricted subset of 2023–2024 was also performed (Figure 5), and although the direction of effects was mostly consistent, in this period of 2023–2024, none of the results reached statistical significance. However, the detailed statistical data—particularly the odds ratios and the relatively wide confidence intervals—suggested that the small sample size might have substantially influenced the results.

The forest plot illustrates the association between different antibiotic prophylaxis regimens and the incidence of surgical site infections (SSIs). Each row represents a comparison between the two treatment groups within a subset of the study population. The effect size is expressed as the odds ratio (OR), calculated as the odds of infection in group 1 divided by the odds in group 2. *p*-values were derived using Fisher’s exact test.

## 3. Discussion

Surgical site infections are among the most serious postoperative complications of instrumented lumbar spine surgery, significantly affecting patient outcomes, prolonging hospital stay, increasing healthcare costs, and impairing functional results [1,2,3]. The results of our study confirm that the type of prophylactic antibiotics is a key factor in preventing SSIs, while the duration of antibiotic administration has a less significant impact on this risk.

Our results clearly support that the choice of prophylactic antibiotics is crucial in preventing infections in instrumented lumbar spine surgeries. The use of cefazolin in both 1 g and 2 g doses resulted in a significantly lower SSI rate than the use of clindamycin, regardless of the duration of antibiotic prophylaxis. This result is confirmed by other previous studies, which emphasize the effectiveness of cefazolin as a first-generation cephalosporin in surgical prophylaxis due to its broad antimicrobial spectrum against skin and soft tissue flora and its favorable side effect profile [1,14,18]. This observation is particularly important because clindamycin was associated with a higher risk of SSI in some cases, particularly in deep and organ infections, confirming that this agent is less effective in these surgical protocols and its use is recommended primarily for patients allergic to beta-lactams [13,19,20].

Longer, 72 h cefazolin prophylaxis showed a slight advantage in preventing SSIs, but this difference was not statistically significant. This is in line with international guidelines and some studies, which recommend a maximum of 24 h of prophylactic antibiotic administration in terms of resistance, side effects, and cost-effectiveness [14,16,21,22,23]. Although some recent studies have shown a positive effect of extended prophylaxis, the evidence is not yet convincing, and individual risk factors must be taken into consideration in clinical decision-making [13,17,18,24,25,26].

It is important to note that in our study, multi-segmental surgeries were associated with higher SSI rates, which is consistent with the greater surgical extent associated with more tissue damage and longer operating times, thus increasing the risk of infection. The majority of infections were deep or systemic infections, which can significantly impair the long-term functional outcomes and quality of life of patients and require complex and costly interventions to treat [1,7,8].

The global spread of antibiotic-resistant bacteria—largely attributable to the inappropriate and unnecessary use of broad-spectrum antibiotics in low-risk situations—reduces the effectiveness of surgical prophylaxis and increases the risk of surgical site infections. Our results, in line with the international literature, show that optimizing surgical prophylaxis—especially the type and dose of antibiotics—is key to reducing the risk of SSIs in spinal surgery. It is notable that although cefazolin proved to be more effective, adherence to the appropriate dosage and timing is essential to maximize its effect and prevent the development of resistance [1,11,14].

Due to the retrospective, observational nature of our study, our study has certain limitations where, for example, in terms of data accuracy and completeness, a uniform assessment of microbiological results was not possible, as several different providers performed the tests during the study period. The patient population came from a single, large university center, so the generalizability of the results may be limited to other institutions or different patient populations. The comparison of the duration of antibiotic prophylaxis only covered a relatively short period between 2023 and 2024, so differences during longer follow-up may not have been apparent. Furthermore, although several relevant risk factors were taken into consideration (e.g., BMI, number of operated segments, diabetes mellitus), other potentially influencing factors, such as smoking, surgical duration, blood loss, or laboratory parameters, were not fully analyzed, which may further modify the SSI risk profile. Further large-scale, randomized, prospective studies are needed to determine the optimal dosage and duration of antibiotic prophylaxis, particularly considering patient-specific risk factors and current antimicrobial guidelines. To address this need, we are planning a comparative study to evaluate single dose versus 72 h prophylaxis regimens in the near future.

## 4. Patients and Methods

### 4.1. Patients Selection

A retrospective review was conducted on a cohort of 824 patients undergoing 915 instrumented lumbar spine surgeries between January 2016 and March 2024 at the Department of Neurosurgery and Neurointervention, Semmelweis University (Budapest, Hungary). All the instrumented lumbar spine surgeries were performed by the same spine surgeon team. All the patients were included in the hospital’s database and were followed up in the electronic medical records. However, patients were excluded if (*i*) they received alternative antibiotic prophylaxis, specifically amoxycillin/clavulanic acid, ceftriaxone, or vancomycin; (*ii*) they were younger than 14 years, because the institution’s profile covers adult care and they do not perform spinal surgery on children under the age of 14; or (*iii*) the patient did not show up for follow-up examinations and data could not be collected in any other way.

### 4.2. Antibiotic Prophylaxis Protocols

Between January 2016 and March 2024, 824 patients who underwent 915 instrumented lumbar spine surgeries received an intravenous antibiotic prophylaxis protocol consisting of the administration of 1 g or 2 g cefazolin 30 min preoperatively, orpatients with allergy to beta-lactams received alternative antibiotics with 600 mg administered clindamycin before the procedure. However, from January 2023 to March 2024, patients received either one shot or prolonged 72 h antibiotic prophylaxis of cefazolin or clindamycin. During this recent period, 174 patients underwent instrumented lumbar spine surgery. Patients were allocated to different prophylaxis durations in a non-randomized, observational manner. Antibiotic duration was selected based on the individual clinical judgment of the operating surgeon. There was no intentional variation between the groups with respect to surgical technique, surgical team, preoperative disinfection protocol, surgical site preparation, or postoperative wound care. Every patient was followed up for at least 1 year.

### 4.3. SSI Definition, Variables Analyzed, and Exclusions

SSIs were classified according to the Center for Disease Control (CDC) guidelines [27]: (i) purulent discharge observed from different layers of the wound (superficial, deep, or intra-organ space, e.g., disk, epidural space, implant); (ii) microorganism detection in a specimen obtained under sterile conditions; (iii) spontaneous or surgically opened wound with at least one of the following symptoms: pain, inflammation (redness, warmth, swelling), or fever (>38 °C); (iv) evidence of abscess by imaging or during surgery. Importantly, a positive microbiological result was not required for diagnosis; cases meeting the clinical or imaging criteria were classified as SSI even if cultures were negative or not performed.

The SSI was categorized as superficial infection if there was one of the following symptoms: purulent drainage from wound; positive wound culture; pain, redness, swelling; diagnosis by surgeon. The SSI was categorized as deep infection if there was one of the following symptoms: purulent drainage from deep aspect of the wound; dehiscence. The SSI was categorized as organ infection if there was infection in the brain, spinal cord, or surrounding cerebrospinal fluid spaces [28].

Apart from the type of prophylaxis used and the type of SSI, the following patient-dependent variables were gathered: age, sex, diabetes mellitus, and body mass index (BMI). The surgery-dependent variables analyzed were minimal invasive spine surgery (MISS) and number of operated segments.

### 4.4. Statistic Analysis

Statistical analysis was carried out in R (version 4.4.3) using the Fisher test and Welch test function to calculate the odd ratio (OR), its confidence interval (CI), and the *p*-value for the univariable analyses. A multivariable ordinal logistic regression model with a cumulative link was also carried out using the clm function of the ordinal R package (version 2023.12.4.1) [29]. Contrasts with Tuckey adjustment were calculated using the emmeans R package (version 1.11.1) [30]. The significance level was 5% [31].

## 5. Conclusions

Our study shows that cefazolin is more effective than clindamycin in preventing infections during prophylaxis for instrumented lumbar spine surgery, regardless of how long the antibiotic is given. Although 72 h cefazolin prophylaxis showed a slight advantage in terms of the infection-free period, the difference was not statistically significant, so single dose or short-term prophylaxis may be slightly preferable in terms of minimizing resistance and side effects. The risk of infection increased in the case of multi-segment surgeries, which may serve as an additional consideration in the design of surgical protocols.

Our results confirm the current guidelines recommending the rapid, adequate, but short-term use of cefazolin, while highlighting the need for an ongoing review of prophylactic antibiotic strategies to minimize infectious complications, ensure patient safety, and promote the efficient use of healthcare resources.

## Figures and Tables

**Table 1:** Characteristics of patients with instrumented lumbar spinal surgery.

Characteristics of the Patients	Non-Infected Cases (807)	SSI Cases (107)	*p*-Value
Age (years), mean (SD ^1^)	63.4 (12.2)	65.5 (11.6)	0.0890 (Welch)
min; 1st qu. ^2^; median; 3rd qu.; max	14.2; 56.6; 65.7; 72.3; 87.6	21.5; 60.6; 67.5; 73.6; 83.6	
Male (%)	330 (40.9%)	36 (33.6%)	0.1724 (Fisher)
Diabetes mellitus (%)	169 (21.0%) (N/A ^3^ = 1)	28 (26.2%)	0.2136 (Fisher)
BMI ^4^, mean (SD) min; 1st qu.; median; 3rd qu.; max	29.1 (5.1) (N/A = 5) 16.7; 25.5; 28.9; 32.4; 48.3	30.9 (6.4) 17.6; 26.7; 30.5; 34.3; 58.2	0.0081 (Welch)
MISS ^5^ (%)	160 (19.8%)	16 (15.0%)	0.2959 (Fisher)
Segments (%)	(N/A = 1)		0.0005 (Fisher)
1–2	698 (86.6%)	71 (66.4%)	
3–7	106 (13.2%)	36 (33.6%)	
≥8	2 (0.2%)	0 (0%)	
SSIs ^6^ (%)			
Superficial SSIs	N/A	30 (28.0%)	
Deep SSIs	N/A	36 (33.6%)	
Organ SSIs	N/A	41 (38.3%)	

^1^ SD: standard deviation; ^2^ qu.: quartile; ^3^ N/A: not available; ^4^ BMI: body mass index; ^5^ MISS: minimal invasive spine surgery; ^6^ SSI: surgical site infection.

**Table 2:** The different prophylactic antibiotic regimens used during the study period.

Antibiotics	Dosage	Duration	Time Interval
Cefazolin	1 g	Single dose	2016–2024
Cefazolin	1 g	72 h	2023–2024
Cefazolin	2 g	Single dose	2016–2024
Cefazolin	2 g	72 h	2023–2024
Clindamycin	0.6 g	Single dose	2016–2024
Clindamycin	0.6 g	72 h	2023–2024

**Table 3:** Association between type of antibiotic prophylaxis regimen (single dose vs. 72 h) and occurrence of surgical site infections (SSIs), including their subtypes.

SSIs	Total n = 253	Single Dose Antibiotic n = 227	72 h Antibiotic n = 26
**None**	**230**	**205**	**25**
		Cefazolin 1 g = 90	Cefazolin 1 g = 6
		Cefazolin 2 g = 106	Cefazolin 2 g = 15
		Clindamycin 0.6 g = 9	Clindamycin 0.6 g = 4
**Superficial**	**2**	**2**	**0**
		Cefazolin 1 g = 2	
**Deep**	**11**	**10**	**1**
		Cefazolin 1 g = 4	Cefazolin 1 g
		Cefazolin 2 g = 6	
**Organ**	**10**	**10**	**0**
		Cefazolin 1 g = 4	
		Cefazolin 2 g = 3	
		Clindamycin 0.6 g = 3	

## Data Availability

The datasets used and/or analyzed during the current study are available from the corresponding author.

## Abbreviations

The following abbreviations are used in this manuscript:

BMI	body mass index
DM	diabetes mellitus
MISS	minimal invasive spine surgery
N/A	not available
OR	odds ratio
qu	quartile
SD	standard deviation
SSI	surgical site infection
